# Supplementary material for: The Influence of Different Forms of Silver on Selected Pathogenic Bacteria
Source: Materials (Basel). 2020 May 23;13(10):2403. doi: 10.3390/ma13102403 (PMC7287713; doi:10.3390/ma13102403)
Supplement: Supplementary file 1 [file materials-13-02403-s001.pdf]

Supplementary material

Table S1. Summary of the changes in signals observed on MALDI-TOF MS spectra recorded for lipids isolated from *E. coli* cells after its incubation with silver ions (Ag^+), tetracycline (TET), ampicillin (AMP), LCLB56 silver (bio)nanpoarticles (LCLB56 AgNPs) and LCLB56 silver (bio)nanpoarticles functionalized with ampicillin (LCLB56 AgNPs/AMP) and tetracycline (LCLB56 AgNPs/TET) as well as native bacteria with names of assigned probable lipids.

	Teoretical m/z	Observed m/z	Native	Ag^+	LCLB56 AgNPs	LCLB56 AgNPs/AMP	LCLB56 AgNPs/TET	AMP	TET	Assigned Lipid	Mode
1	215.1160	215.096	+					+	+	[LPG(12:0) + 2H]$^{2+}$	+
2	248.1406	248.139		+	+					[LPG(17:0) − 2H]$^{2-}$	−
3	284.1865	284.186		+	+	+	+			[LPG(22:1) + 2H]$^{2+}$	+
4	285.1943	285.175		+	+	+	+			[LPG(22:0) + 2H]$^{2+}$	+
5	303.2321	303.232		+						[PG(O-47:3) − 3H]$^{3-}$	−
6	305.1747	305.196	+					+		[PG(23:0(OH)) − 2H]$^{2-}$	−
7	312.1825	312.185							+	[PG(24:0(OH)) − 2H]$^{2-}$	−
8	322.8920	322.876			+		+	+	+	[PE(52:8(OH)) − 3H]$^{3-}$	−
9	328.9077	328.895		+	+	+	+			[PE(53:6(OH)) − 3H]$^{3-}$	−
10	330.8920	330.889		+	+	+	+			[PE(54:10(OH)) − 3H]$^{3-}$	−
11	428.3273	428.323		+	+	+	+			[PG(P-43:3) + 2H]$^{2+}$	+
12	430.3430	430.362		+	+	+	+			[PG(P-43:1) + 2H]$^{2+}$	+
13	346.7008	346.685		+	+					[PE(32:6(OH)) − 2H]$^{2-}$	−
14	373.9701	373.97							+	[PG(61:2) − 3H]$^{3-}$	−
15	378.3261	378.323		+	+					[PE(64:1) − 3H]$^{3-}$	−
16	427.9365	427.936		+	+	+	+			[CL(60:5) − 3H]$^{3-}$	−
17	429.9521	429.937		+	+	+	+			[CL(60:2) − 3H]$^{3-}$	−
18	436.8129	436.819		+	+	+	+			[PE(46:6) − 2H]$^{2-}$	−
19	550.3878	550.407		+	+	+	+		+	[LPE(23:0) − H]$^{-}$	−
20	554.3827	554.412		+						[LPE(O-20:0) + OAc]$^{-}$	−
21	566.3464	566.373		+	+	+	+			[PE(22:0(OH)) − H]$^{-}$	−
22	576.4824	576.483	+					+	+	[PG(63:1) − 2H]$^{2-}$	−
23	580.3409	580.339		+	+	+				[LPE(26:6) − H]$^{-}$	−
24	582.3565	582.342		+	+	+				[LPE(26:5) − H]$^{-}$	−

25	584.3722	584.328	+	+	+		[LPE(26:4) − H]$^-$	−
26	594.3332	594.299	+	+	+	+	[LPE(24:3) + Cl]$^-$	−
27	598.3362	598.308	+	+	+	+	[PE(20:0(OH)) + OAc]$^-$	−

*According to LIPID MAPS® Lipidomics Gateway database.

Table S2. Summary of the changes in signals observed on MALDI-TOF MS spectra recorded for lipids isolated from *S. aureus* cells after its incubation with silver ions (Ag^+), tetracycline (TET), ampicillin (AMP), LCLB56 silver (bio)nanpoarticles (LCLB56 AgNPs) and LCLB56 silver (bio)nanpoarticles functionalized with ampicillin (LCLB56 AgNPs/AMP) and tetracycline (LCLB56 AgNPs/TET) as well as native bacteria with names of assigned probable lipids.

	Teoretical m/z	Observed m/z	Native	Ag^+	LCLB56 AgNPs	LCLB56 AgNPs/AMP	LCLB56 AgNPs/TET	AMP	TET	Assigned Lipid	Mode
1	284.1865	284.189		+	+	+	+	+	+	$[LPG(22:1) + 2H]^{2+}$	+
2	285.1943	285.187		+	+	+	+	+	+	$[LPG(22:0) + 2H]^{2+}$	+
3	303.2321	303.247		+	+	+	+	+	+	$[PG(O\text{-}47:3) - 3H]^{3-}$	−
4	312.1502	312.143		+	+	+	+			$[PG(22:2) + 2Na]^{2+}$	+
5	316.2684	316.251		+	+	+	+	+	+	$[DG(37:4) + 2H]^{2+}$	+
6	328.2684	328.252			+	+	+	+	+	$[DG(39:6) + 2H]^{2+}$	+
7	391.2725	391.275				+		+	+	$[PG(O\text{-}38:6) + 2H]^{2+}$	+
8	409.2009	409.204	+							$[PI(24:9) - 2H]^{2-}$	−
9	416.3625	416.359		+	+	+	+	+	+	$[DG(O\text{-}49:3) + 2Na]^{2+}$	+
10	492.2248	429.260		+	+	+	+	+	+	$[PG(12:0) + H]^{+}$	+
11	443.3508	443.373				+				$[PG(O\text{-}45:3) + 2H]^{2+}$	+
12	453.2976	453.317		+	+	+	+	+	+	$[LPG(O\text{-}16:0) + H - H_2O]^{+}$	+
13	453.4302	453.477		+	+	+	+	+	+	$[DG(O\text{-}26:0) + H - H_2O]^{+}$	+
14	460.4093	460.425		+	+	+	+	+	+	$[TG(O\text{-}58:7) + 2H]^{2+}$	+
15	465.9834	465.969		+						$[CL(68:4) - 3H]^{3-}$	−
16	472.4562	472.473		+	+	+	+	+	+	$[TG(O\text{-}59:2) + 2H]^{2+}$	+
17	500.3754	500.368				+			+	$[PG(P\text{-}54:6) - 2H]^{2-}$	−
18	550.4198	550.431		+	+	+	+	+	+	$[PG(60:6) - 2H]^{2-}$	−
19	566.3859	566.394		+	+	+	+	+	+	$[PG(62:12(OH)) - 2H]^{2-}$	−
20	757.4650	757.423		+	+	+	+	+	+	$[PG(34:5(OH)) + H]^{+}$	+
21	1701.3347	1701.36	+	+	+	+	+	+		$[CL(88:3) + NH_4]^{+}$	+

*According to LIPID MAPS® Lipidomics Gateway database.

Table S3. Summary of the changes in signals observed on MALDI-TOF MS spectra recorded for lipids isolated from *S. epidermidis* cells after its incubation with silver ions (Ag^+), tetracycline (TET), ampicillin (AMP), LCLB56 silver (bio)nanpoarticles (LCLB56 AgNPs) and LCLB56 silver (bio)nanpoarticles functionalized with ampicillin (LCLB56 AgNPs/AMP) and tetracycline (LCLB56 AgNPs/TET) as well as native bacteria with names of assigned probable lipids.

	Teoretical m/z	Observed m/z	Native	Ag^+	LCLB56 AgNPs	LCLB56 AgNPs/AMP	LCLB56 AgNPs/TET	AMP	TET	Assigned Lipid	Mode
1	286.4797	286.476		+	+					[PIP(30:0) – 3H]$^{3-}$	–
2	312.1537	312.153		+				+	+	[PG(26:6) – 2H]$^{2-}$	–
3	316.2684	316.253		+	+	+	+			[DG(37:4) + 2H]$^{2+}$	+
4	328.2684	328.255		+	+	+	+			[DG(39:6) + 2H]$^{2+}$	+
5	330.2359	330.235		+	+	+	+			[LPG(O-30:4) + 2H]$^{2+}$	+
6	368.5587	368.563		+		+		+		[PI(55:12) – 3H]$^{3-}$	–
7	385.3389	385.336		+	+	+	+			[DG(47:5) + 2H]$^{2+}$	+
8	390.3942	390.433		+	+		+			[MG(O-20:0) + NH_4]$^{+}$	+
9	391.1891	391.189	+	+	+			+		[LPA(15:2) – H]$^{-}$	–
10	406.2320	406.234		+						[PG(40:10) – 2H]$^{2-}$	–
11	416.3273	416.351		+	+	+	+			[PG(P-41:1) + 2H]$^{2+}$	+
12	427.9365	427.937		+	+	+	+			[CL(60:5) – 3H]$^{3-}$	–
13	429.2248	429.946		+	+	+	+	+	+	[PG(12:0) + H]$^{+}$	–
14	430.4093	430.386		+	+	+	+			[TG(O-53:2) + 2H]$^{2+}$	+
15	439.9999	440.029		+		+				[PI(68:4;O) – 3H]$^{3-}$	–
16	443.3508	443.369		+						[PG(O-45:3) + 2H]$^{2+}$	+
17	451.4328	451.445		+	+	+	+			[TG(O-56:2) + 2H]$^{2+}$	+
18	453.2976	453.301		+	+	+	+			[LPG(O-16:0) + H – H_2O]$^{+}$	+
19	453.4484	453.464		+	+	+	+			[TG(O-56:0) + 2H]$^{2+}$	+
20	460.4093	460.425		+	+	+	+			[TG(O-58:7) + 2H]$^{2+}$	+
21	488.3848	488.385	+					+	+	[PG(49:1(OH)) + 2H]$^{2+}$	+
22	497.4641	497.472		+	+	+	+			[TG(O-63:5) + 2H]$^{2+}$	+
23	500.3390	500.348		+	+		+			[PG(52:8(OH)) – 2H]$^{2-}$	–
24	518.3616	518.449			+		+			[LPC(O-19:2) – H]$^{-}$	–
25	553.3875	553.483		+						[LPG(O-22:0) – H]$^{-}$	–
26	554.4041	554.409			+					[PG(61:9) – 2H]$^{2-}$	–
27	566.3859	566.376		+	+	+	+			[PG(62:12(OH)) – 2H]$^{2-}$	–

28	580.3409	580.345		+	+		+			$[LPC(23:6) - H]^-$	−
29	582.3565	582.363		+	+					$[LPE(26:5) - H]^-$	−
30	592.4955	592.503	+					+		$[PG(64:0(OH)) - 2H]^{2-}$	−
31	643.3969	643.438	+					+		$[LPG(28:5) + H]^+$	+
32	644.4230	644.441						+	+	$[CL(60:5) + 2H]^{2+}$	+
33	1449.9795	1449.938		+	+	+	+			$[CL(72:8) + H]^+$	+

*According to LIPID MAPS® Lipidomics Gateway database

Table S4. Summary of the changes in signals observed on MALDI-TOF MS spectra recorded for lipids isolated from *K. pneumoniae* cells after its incubation with silver ions (Ag^+), tetracycline (TET), ampicillin (AMP), LCLB56 silver (bio)nanpoarticles (LCLB56 AgNPs) and LCLB56 silver (bio)nanpoarticles functionalized with ampicillin (LCLB56 AgNPs/AMP) and tetracycline (LCLB56 AgNPs/TET) as well as native bacteria with names of assigned probable lipids.

	Teoretical m/z	Observed m/z	Native	Ag^+	LCLB56 AgNPs	LCLB56 AgNPs/AMP	LCLB56 AgNPs/TET	AMP	TET	Assigned Lipid	Mode
1	226.1628	226.142		+					+	[LPA(P-20:0) + 2H]$^{2+}$	+
2	242.1289	242.115			+	+	+			[LPA(22:6) + 2H]$^{2+}$	+
3	247.1938	247.19				+	+			[PA(O-40:1) − 3H]$^{3-}$	−
4	248.1588	248.153			+	+	+			[LPG(O-18:0) − 2H]$^{2-}$	−
5	255.1484	255.146			+	+	+			[LPG(18:0) − 2H]$^{2-}$	−
6	257.8137	257.816		+	+			+	+	[PG(37:8) − 3H]$^{3-}$	−
7	276.2553	276.27		+						[DG(P-18:1) + 2H]$^{2+}$	+
8	284.1759	284.17		+	+	+	+			[LPA(28:6) + 2H]$^{2+}$	+
9	303.2136	303.232			+					[PA(O-29:0(OH)) − 2H]$^{2-}$	−
10	312.1218	312.103	+			+	+			[PC(O-4:0) − CH_3]$^{-}$	−
11	314.1512	314.145	+				+			[PG(25:5;O) − 2H]$^{2-}$	−
12	330.8920	330.893			+					[PE(54:10(OH)) − 3H]$^{3-}$	−
13	340.9077	340.902		+	+	+	+			[PC(53:9(OH)) − 3H]$^{3-}$	−
14	342.8990	342.883		+	+	+	+			[PS(53:7(OH)) − 3H]$^{3-}$	−
15	344.6958	344.689		+		+				[PS(28:2(OH)) − 2H]$^{2-}$	−
16	346.7008	346.689			+					[PE(32:6(OH)) − 2H]$^{2-}$	−
17	353.5917	353.59	+		+	+	+	+	+	[PE(60:10) − 3H]$^{3-}$	−
18	354.7529	354.77		+	+					[LPC(32:4) − 2H]$^{2-}$	−
19	360.5579	360.554			+	+	+			[PIP(46:1) − 3H]$^{3-}$	−
20	368.5829	368.566				+	+			[PG(60:11;O) − 3H]$^{3-}$	−
21	373.9580	373.96	+							[PG(60:3(OH)) − 3H]$^{3-}$	−
22	391.1709	391.177		+				+		[LPIP(26:4) − 2H]$^{2-}$	−
23	391.2244	391.261		+				+	+	[LPA(16:1)+H − H_2O]$^{+}$	+
24	393.2893	393.296			+					[PA(41:1(OH)) − 2H]$^{2-}$	−
25	394.9859	394.991	+					+	+	[PC(64:5(OH)) − 3H]$^{3-}$	−
26	398.2087	398.186		+	+	+		+		[PI(32:6) − 2H]$^{2-}$	−
27	398.9738	398.977							+	[PS(66:6) − 3H]$^{3-}$	−

28	406.2684	406.265				+	+			[PA(45:8) − 2H]$^{2-}$	−
29	409.1815	409.161			+			+	+	[PIP(26:1(OH)) − 2H]$^{2-}$	−
30	411.2153	411.199					+			[LPG(O-12:1) − H]$^{-}$	−
31	422.1893	422.174					+			[PIP(28:2(OH)) − 2H]$^{2-}$	−
32	427.9365	427.939			+	+	+			[CL(60:5) − 3H]$^{3-}$	−
33	429.2248	429.942			+	+	+			[PG(12:0) + H]$^{+}$	−
34	430.3441	430.346			+	+	+			[TG(53:9) + 2H]$^{2+}$	+
35	436.8311	436.832			+	+	+	+		[PC(O-44:6) − 2H]$^{2-}$	−
36	450.8755	450.882				+	+			[PC(O-44:0(OH)) − 2H]$^{2-}$	−
37	466.8235	466.824			+	+	+			[PS(47:5) − 2H]$^{2-}$	−
38	467.8859	467.903				+	+			[PC(O-48:3) − 2H]$^{2-}$	−
39	498.6553	498.644	+		+	+				[CL(76:11) − 3H]$^{3-}$	−
40	499.4797	499.48			+	+	+			[DG(63:3) + 2H]$^{2+}$	+
41	500.4223	500.419			+	+	+			[PG(O-53:0) − 2H]$^{2-}$	−
42	501.3457	501.347			+	+	+			[PG(52:9(OH)) + 2H]$^{2+}$	+
43	503.3496	503.347			+	+	+			[LPA(24:1) + H − H_2O]$^{+}$	+
44	514.4198	514.421	+		+	+	+			[PG(54:0) − 2H]$^{2-}$	−
45	515.3343	515.387			+	+	+			[LPG(O-18:0;O) + H]$^{+}$	+
46	516.4067	516.414			+	+	+			[PA(59:4(OH)) − 2H]$^{2-}$	−
47	517.3876	517.384			+	+	+			[PI(48:1) + 2H]$^{2+}$	+
48	531.4032	531.403			+	+	+			[PI(O-50:2(OH)) + 2H]$^{2+}$	+
49	533.4007	533.4			+	+	+			[PI(49:0(OH)) + 2H]$^{2+}$	+
50	559.4345	559.435			+	+	+			[PI(54:1) + 2H]$^{2+}$	+
51	564.3671	564.373				+				[PE(23:0) − H]$^{-}$	−
52	566.3464	566.378			+	+	+			[LPS(21:0) − H]$^{-}$	−
53	580.3409	580.343			+	+	+			[LPE(26:6) − H]$^{-}$	−
54	582.3565	582.346			+	+	+			[LPE(26:5) − H]$^{-}$	−
55	590.4980	590.516	+				+	+	+	[PG(65:1) − 2H]$^{2-}$	−
56	594.3413	594.304			+	+				[PS(22:0) − H]$^{-}$	−
57	598.3151	598.31			+	+	+			[LPS(24:5) − H]$^{-}$	−
58	602.4798	602.48	+					+	+	[PG(66:4(OH)) − 2H]$^{2-}$	−
59	843.7212	843.756	+	+	+			+	+	[PA(O-47:0) − H]$^{-}$	−

60	865.7056	865.718		+	+			[PA(O-49:3) − H]⁻	−
61	881.7369	881.732		+		+	+	[PA(O-50:2) − H]⁻	−
62	891.7060	891.717	+					[PG(O-44:0(OH)) − H]⁻	−
63	1076.8264	1076.844		+	+	+	+	[PS(57:4) − H]⁻	−

*According to LIPID MAPS® Lipidomics Gateway database.

Table S5. Summary of the changes in signals observed on MALDI-TOF MS spectra recorded for lipids isolated from *P. aeruginosa* cells after its incubation with silver ions (Ag^+), tetracycline (TET), ampicillin (AMP), LCLB56 silver (bio)nanpoarticles (LCLB56 AgNPs) and LCLB56 silver (bio)nanpoarticles functionalized with ampicillin (LCLB56 AgNPs/AMP) and tetracycline (LCLB56 AgNPs/TET) as well as native bacteria with names of assigned probable lipids.

	Teoretical m/z	Observed m/z	Native	Ag^+	LCLB56 AgNPs	LCLB56 AgNPs/AMP	LCLB56 AgNPs/TET	AMP	TET	Assigned ipid	Mode
1	284.1865	284.189		+	+	+	+	+	+	[LPG(22:1) + 2H]$^{2+}$	+
2	285.5169	285.52		+	+	+	+	+	+	[PE(44:8(OH)) − 3H]$^{3-}$	−
3	285.1943	285.183		+	+	+	+	+	+	[LPG(22:0) + 2H]$^{2+}$	+
4	298.2021	298.214		+	+	+	+	+	+	[LPG(24:1) + 2H]$^{2+}$	+
5	307.1797	307.168		+	+	+	+		+	[LPG(26:4) − 2H]$^{2-}$	−
6	322.9163	322.919						+	+	[PE(P-54:6) − 3H]$^{3-}$	−
7	328.1883	328.236			+	+			+	[PC(O-5:0) + H]$^{+}$	+
8	328.8885	328.893		+	+	+		+	+	[PC(52:12) − 3H]$^{3-}$	−
9	391.2519	391.247						+	+	[PG(O-33:1(OH)) + 2Na]$^{2+}$	+
10	427.9365	427.937		+	+	+	+	+	+	[CL(60:5) − 3H]$^{3-}$	−
11	429.2248	429.944		+	+	+	+	+	+	[PG(12:0) + H]$^{+}$	−
12	436.8311	436.832		+	+	+	+	+	+	[PC(O-44:6) − 2H]$^{2-}$	−
13	550.3878	550.404		+	+	+	+	+	+	[PC(O-21:0) − CH_3]$^{-}$	−
14	582.3332	582.346		+	+	+	+	+		[PC(O-20:2) + Cl]$^{-}$	−
15	594.3332	594.301		+	+	+	+	+	+	[LPE(24:3) + Cl]$^{-}$	−
16	598.3281	598.312		+	+	+	+	+	+	[PC(20:1) + Cl]$^{-}$	−
17	1048.8315	1048.79				+				[PE(57:4(OH)) − H]$^{-}$	−
18	1070.8159	1070.796	+	+	+	+				[PE(59:7(OH)) − H]$^{-}$	−
19	1503.1000	1503.064		+			+		+	[CL(74:4) + NH_4]$^{+}$	+

*According to LIPID MAPS® Lipidomics Gateway database.

Table S6. Summary of the changes in signals observed on MALDI-TOF MS spectra recorded for proteins isolated from *E. coli* cells after its incubation with silver ions (Ag^+), tetracycline (TET), ampicillin (AMP), LCLB56 silver (bio)nanpoarticles (LCLB56 AgNPs) and LCLB56 silver (bio)nanpoarticles functionalized with ampicillin (LCLB56 AgNPs/AMP) and tetracycline (LCLB56 AgNPs/TET) as well as native bacteria with names of assigned probable proteins.

Escherichia coli								
**m/z**	**Native**	**Ag^+**	**LCLB56 AgNPs**	**LCLB56 AgNPs/AMP**	**LCLB56 AgNPs/TET**	**AMP**	**TET**	**Proposed Protein Name**
2205						+		Phage tail protein
2327							+	Type IV secretion protein Rhs
2341	+							RNA helicase
2409						+		Phage tail protein
2834	+	+	+	+	+	+		Aspartate 1-decarboxylase
3075	+		+	+	+	+	+	Small toxic protein shoB
3253		+	+	+	+			Universal stress protein UspG
3404		+	+	+	+			DNA gyrase inhibitor
3411		+	+	+	+			DNA-binding transcriptional repressor LrhA
3793	+							Thiosulfate reductase cytochrome B subunit
4060	+					+	+	Involved in lipopolysaccharide biosynthesis
4466			+					StbA family protein
4468			+					Ribonucleotide reductase transcriptional regulator
4578					+			Lysis protein
4708			+					Transposase
5488					+			Copper resistance protein
6550	+				−	+		Ribosomal large subunit pseudouridine synthase F
6808	+	+	+	+	+			Arc family DNA-binding protein
7964							+	Holin protein
9711			+	+	+			Lysozyme
9714						+		Killer protein

*According to UniProt database.

Table S7. Summary of the changes in signals observed on MALDI-TOF MS spectra recorded for proteins isolated from *S. aureus* cells after its incubation with silver ions (Ag^{+}), tetracycline (TET), ampicillin (AMP), LCLB56 silver (bio)nanpoarticles (LCLB56 AgNPs) and LCLB56 silver (bio)nanpoarticles functionalized with ampicillin (LCLB56 AgNPs/AMP) and tetracycline (LCLB56 AgNPs/TET) as well as native bacteria with names of assigned probable proteins.

	Staphylococcus aureus								
	**m/z**	**Native**	**Ag^{+}**	**LCLB56 AgNPs**	**LCLB56 AgNPs/AMP**	**LCLB56 AgNPs/TET**	**AMP**	**TET**	**Proposed Protein Name**
1	2218			+		+			Uncharacterized protein
2	2280	+	+		+	+			Uncharacterized protein
3	2611			+					G6PD_N domain-containing protein
4	2650	+	+	+	+	+	+		Uncharacterized protein
5	2696		+			+	+	+	Uncharacterized protein
6	2705				+				Transposon DNA-invertase
7	2785			+		+			2-deoxyribose-5-phosphate aldolase
8	2823	+	+	+	+	+	+		Peptide deformylase
9	3183	+					+	+	GNAT family N-acetyltransferase
10	3475	+			+				Uncharacterized protein
11	3784	+	+	+	+		+		Diaminopimelate decarboxylase
12	3792	+	+	+	+		+		2,3-cyclic-nucleotide 2-phosphodiesterase
13	3837				+				Imidazole glycerol phosphate synthase subunit HisF
14	3840			+					Deoxyribose-phosphate aldolase
15	4291			+					50S ribosomal protein L36
16	4318					+			Terminase, superantigen-encoding pathogenicity islands SaPI
17	4320	+	+				+	+	Beta-class phenol-soluble modulin

18	4324			+					Uncharacterized protein
19	4519		+	+	+	+			Uncharacterized protein
20	4735					+			PfkB family carbohydrate kinase
21	4764	+							Amino acid permease
22	4862							+	Antibacterial protein 3
23	4867	+			+				Uncharacterized protein
24	4871							+	50S ribosomal protein L19
25	4877	+							Uncharacterized protein
26	5304	+	+	+	+	+	+		Peptide chain release factor 2
27	5319				+		+		Pathogenicity island protein
28	5411				+				SceD-like transglycosylase, biomarker for vancomycin-intermediate strains
29	5632				+				Pyruvate carboxylase
30	5900					+			Transposase-like protein
31	5932	+	+	+	+	+			50S ribosomal protein L33
32	6507					+			DNA-binding protein
33	6531				+				Uncharacterized protein
34	6709			+					Esterase
35	6735			+					Intercellular adhesion protein D
36	6779		+	+	+				Uncharacterized protein
37	6995			+	+				DNA binding domain, excisionase family
38	7010			+					HTH cro/C1-type domain-containing protein
39	7021			+					Thiol peroxidase, Bcp-type
40	7047			+					Nitrogen fixation protein NifR
41	7139			+					Cation diffusion facilitator family transporter
42	7292			+					DNA-binding protein
43	7567	+	+	+	+		+		Replication protein

44	7579		+			+		ABC transporter ATP-binding protein uup
45	7582	+					+	Serine hydroxymethyltransferase
46	7680			+				Transcriptional regulator
47	7713			+		+		Prolipoprotein diacylglyceryl transferase
48	7741			+				PHB domain-containing protein
49	7785			+				Transcriptional regulator
50	8998				+			Transposase
51	9467					+		Aspartate aminotransferase
52	9526	+						Enterotoxin B
53	9734				+			50S ribosomal protein L7ae-like protein
54	9758				+			50S ribosomal protein L21
55	9797				+			50S ribosomal protein L31 type B
56	9840				+			Uncharacterized protein
57	9875		+	+				Tryptophan synthase subunit beta
58	9881					+		Uncharacterized protein
59	9911					+		RecA protein
60	9947			+				Reverse transcriptase-like protein
61	9988			+				LPXTG-motif protein cell wall anchor domain protein

*According to UniProt database.

Table S8. Summary of the changes in signals observed on MALDI-TOF MS spectra recorded for proteins isolated from *S. epidermidis* cells after its incubation with silver ions (Ag^{+}), tetracycline (TET), ampicillin (AMP), LCLB56 silver (bio)nanpoarticles (LCLB56 AgNPs) and LCLB56 silver (bio)nanpoarticles functionalized with ampicillin (LCLB56 AgNPs/AMP) and tetracycline (LCLB56 AgNPs/TET) as well as native bacteria with names of assigned probable proteins.

					Staphylococcus epidermidis				
	**m/z**	**Native**	**Ag^{+}**	**LCLB56 AgNPs**	**LCLB56 AgNPs/AMP**	**LCLB56 AgNPs/TE T**	**AM P**	**TET**	**Proposed Protein Name**
1	2242	+	+					+	Membrane protein YoeI
2	2995	+	+				+	+	PTS N-acetylgalactosamine transporter subunit IID
3	3209	+	+	+	+	+	+		Transcriptional regulator BetI
4	3612		+						Uncharacterized protein
5	3615	+					+	+	Uncharacterized protein
6	3699	+	+				+	+	Uncharacterized protein
7	3879			+	+	+			Uncharacterized protein
8	3889	+	+				+	+	Transcriptional regulator
9	4112	+	+	+			+	+	Uncharacterized protein
10	4305			+	+	+	+	+	50S ribosomal protein L36
11	4342		+				+	+	Xanthine phosphoribosyltransferase
12	4353	+	+				+	+	Uncharacterized protein
13	4414		+				+		Uncharacterized protein
14	4950	+							Transcriptional regulator CynR
15	5024				+				Uncharacterized protein
16	5054		+					+	XRE-family like protein
17	5067		+						Uncharacterized protein
18	5829	+	+			+	+		Uncharacterized protein
19	5946		+	+	+	+	+	+	50S ribosomal protein L33
20	6321			+					Uncharacterized protein
21	6516			+	+	+			50S ribosomal protein L32
22	6534			+					Dihydrolipoamide acetyltransferase

23	7777	+	+			+	+	+	Uncharacterized protein
24	10429		+	+	+	+	+	+	Formate dehydrogenase

*According to UniProt database.

Table S9. Summary of the changes in signals observed on MALDI-TOF MS spectra recorded for proteins isolated from *K. pneumoniae* cells after its incubation with silver ions (Ag^{+}), tetracycline (TET), ampicillin (AMP), LCLB56 silver (bio)nanpoarticles (LCLB56 AgNPs) and LCLB56 silver (bio)nanpoarticles functionalized with ampicillin (LCLB56 AgNPs/AMP) and tetracycline (LCLB56 AgNPs/TET) as well as native bacteria with names of assigned probable proteins.

Klebsiella pneumoniae									
	**m/z**	**Native**	**Ag^{+}**	**LCLB56 AgNPs**	**LCLB56 AgNPs/AMP**	**LCLB56 AgNPs/TET**	**AMP**	**TET**	**Proposed Protein Name**
1	2061					+			DUF1471 domain-containing protein
2	2137						+		Type I toxin-antitoxin system Ibs family toxin
3	2264						+		IlvGEDA operon leader peptide
4	2327						+		Small toxic polypeptide
5	2721						+	+	SecA regulator SecM
6	2789	+	+	+			+	+	Uncharacterized protein
7	3496			+					Complement resistance protein TraT
8	3580	+	+	+			+	+	AP endonuclease
9	3839			+					Cobalt-precorrin-6y C15-methyltransferase
10	3857				+				Uncharacterized protein
11	3879			+	+	+			Transposase
12	3892			+					Membrane protein YfcA
13	3912				+				Heat-shock protein
14	4143					+			Cold-shock protein
15	4155	+	+	+	+	+		+	Long-chain-fatty-acid--CoA ligase
16	4185	+	+	+			+	+	Putative phosphatase YqaB
17	4306				+	+			Methyltransferase
18	4324			+					NmrA family protein
19	4810			+					Entericidin B
20	5436	+	+						NADH-ubiquinone oxidoreductase subunit H

21	5516			+					IS3 family transposase
22	5658						+		Phosphatidylglycerophosphatase B
23	5929			+					Lipoate-protein ligase A
24	6137			+					DNA repair protein RadA
25	6152			+		+	+		LLM class flavin-dependent oxidoreductase
26	6509				+				Membrane protein
27	6612						+		Mg(2+) transport ATPase, P-type
28	6678				+				ATP synthase subunit A
29	6993			+					Transcriptional regulator
30	7008			+					Cell division protein FtsA
31	7029			+					Arsenic efflux pump protein
32	7678			+					Membrane protein
33	8369	+	+	+		+	+	+	Nitrate reductase
34	8603	+					+		ABC transporter permease
35	9618			+					Klebicin B immunity
36	9883				+	+			Urease subunit alpha
37	10076		+	+		+			Benzoate 1,2-dioxygenase
38	10079	+					+	+	Hydrolase

*According to UniProt database.

Table S10. Summary of the changes in signals observed on MALDI-TOF MS spectra recorded for proteins isolated from *P. aeruginosa* cells after its incubation with silver ions (Ag^{+}), tetracycline (TET), ampicillin (AMP), LCLB56 silver (bio)nanpoarticles (LCLB56 AgNPs) and LCLB56 silver (bio)nanpoarticles functionalized with ampicillin (LCLB56 AgNPs/AMP) and tetracycline (LCLB56 AgNPs/TET) as well as native bacteria with names of assigned probable proteins.

Pseudomonas aeruginosa										
	**m/z**	**Native**	**Ag^{+}**	**LCLB56 AgNPs**	**LCLB56 AgNPs/AMP**	**LCLB56 AgNPs/TET**	**AMP**	**TET**	**Proposed Protein Name**
1	2734	+	+	-	-	+	+	+	Cryptic plasmid protein A
2	2965			+	+				Uncharacterized protein
3	3168			+	+				Thermonuclease family protein
4	3182			+					Mercury resistance protein
5	3366			+	+				Uncharacterized protein
6	3369							+	ADP-ribosyl-[dinitrogen reductase] hydrolase
7	3409	+	+	-	-	+	+	+	Uncharacterized protein
8	3438			+	+				Uncharacterized protein
9	3601	+	+	+	-	+	+	+	Uncharacterized protein
10	3642		+				+		Phage capsid protein
11	3646			+	+				3-isopropylmalate dehydratase
12	3656	+	+	+	-	+	+	-	Uncharacterized protein
13	3798	+	+				+	+	30S ribosomal protein S20
14	3806	+	+				+	+	NADPH:quinone oxidoreductase family protein
15	3812	+	+				+	+	Mg_chelatase_C domain-containing protein
16	3840			+	+				GNAT family N-acetyltransferase
17	3856			+	+	+			Periplasmic inhibitor/zinc-resistance associated protein
18	3892			+	+				Uncharacterized protein
19	3905			+	+				Protein-disulfide isomerase
20	4305					+			ATP-binding protein

21	4325			+	+				Host-nuclease inhibitor protein Gam
22	4333						+		Rhodanese-like domain-containing protein
23	4496		+					+	TIGR01459 family HAD-type hydrolase
24	4512	+	+				+		Short chain dehydrogenase
25	4525	+							MFS transporter
26	4810			+	+				Glycosyltransferase
27	5273	+	+	-	-	-	+	+	Monovalent cation/H+ antiporter subunit F
28	5301							+	Colicin_immun domain-containing protein
29	5313			+	+				Uncharacterized protein
30	5331			+	+				Metal dependent phosphohydrolase
31	5450	+	+				+		Uncharacterized protein
32	5929			+	+				C4-dicarboxylate transporter, DctQ subunit
33	6336			+	+				Two-component sensor
34	6625		+			+			Transcriptional regulator
35	6732			+	+				GntR family transcriptional regulator
36	6876			+	+				IS5/IS1182 family transposase
37	6993			+	+				DUF1289 domain-containing protein
38	7008			+	+				Uncharacterized protein
39	7025	+	+	-	-	+	+	+	Cytochrome c protein
40	7290			+	+				Peptidyl-prolyl cis-trans isomerase
41	7297							+	50S ribosomal protein L35
42	7328	+	+	+	-	+	+	+	Copper chaperone

43	7678			+	+				ATP synthase subunit c
44	7694			+	+				Uncharacterized protein
45	7713			+	+	+			Carbamate kinase
46	7784			+	+				DNA-binding protein
47	7810			+	+				Beta-ketoacyl-ACP synthase II
48	7823			+	+				ABC transporter ATP-binding protein
49	8565	+							KORA domain-containing protein
50	8688					+	+	+	Uncharacterized protein
51	8785	+	+	-	-	+	+	+	Uncharacterized protein
52	9618			+	+				DNA-binding protein
53	9849						+	+	HU family DNA-binding protein
54	9883					+			DNA-binding protein
55	9945			+	+				ORF_5
56	12137		+						Murein biosynthesis integral membrane protein MurJ
57	12298		+						Sulfurtransferase

*According to UniProt database.

Table S11. Summary of the proteins common for different bacteria treated with silver ions (Ag^{+}), tetracycline (TET), ampicillin (AMP), LCLB56 silver (bio)nanpoarticles (LCLB56 AgNPs) and LCLB56 silver (bio)nanpoarticles functionalized with ampicillin (LCLB56 AgNPs/AMP) and tetracycline (LCLB56 AgNPs/TET) as well as native bacteria.

**Protein**	**Baceria strain**	**Native**	**Ag^{+}**	**LCLB56 AgNPs**	**LCLB56 AgNPs/AMP**	**LCLB56 AgNPs/TET**	**AMP**	**TET**
Transposase proteins family	*E.coli*			+				
	S. aureus				+	+		
	P. aeruginosa			+	+			
	K. pneumoniae			+	+	+		
DNA binding protein	*E.coli*	+	+	+	+	+		
	S. aureus					+		
	P. aeruginosa			+	+	+	+	+
ABC Transporter ATP-binding protein	*S. aureus*		+			+		
	P. aeruginosa			+	+			
ATP synthase subunit	*P. aeruginosa*			+	+			
	K. pneumoniae				+			
Transcriptional regulator	*P. aeruginosa*		+			+		
	K. pneumoniae			+				
	S. aureus			+				
	S. epidermidis	+	+				+	+
50S ribosomal protein L36	*S. aureus*			+				
	S. epidermidis			+	+	+	+	+
50S ribosomal protein L33	*S. aureus*	+	+	+	+	+		
	S. epidermidis		+	+	+	+	+	+

*According to UniProt database.
